# Implementation of an Algorithm to Prevent Chatter Vibration in a CNC System

**DOI:** 10.3390/ma12193193

**Published:** 2019-09-29

**Authors:** Marcin Jasiewicz, Karol Miądlicki

**Affiliations:** Department of Mechanical Engineering and Mechatronics, West Pomeranian University of Technology, Szczecin, al. Piastów 19, 70–310 Szczecin, Poland

**Keywords:** chatter, CNC control, machining assistance, machining stability, receptance coupling, turning, operator, HMI

## Abstract

Machining of shafts characterized by a high compliance is difficult due to the occurrence of self-excited chatter vibrations. It is possible to limit their occurrence through the appropriate selection of technological parameters. For a proper selection of these parameters it is necessary to know the dynamic properties of the machine–tool–workpiece. This study proposes an approach through which these properties can be determined as a result of the synthesis of the dynamic properties of the system, using the receptance coupling method. Knowledge of these properties allows us to select the technological parameters of the lathe using the assistance system integrated into the CNC (Computerized Numerical Control). The final section of this work presents the experimental validation of the assistant and proposed procedures.

## 1. Introduction

The occurrence of self-excited chatter vibrations is one of the main reasons for limiting the efficiency of machining. Performing cutting with chatter is unacceptable due to the traces left on the workpiece surface (so-called chatter marks), the noise, the risk of damage to the tool, or even the machine tool subassemblies [1]. The problem of chatter vibrations is particularly troublesome while machining the workpieces with low stiffness [2,3]. An example of such machining can be the turning of slender shafts.

Research work on limiting the occurrence of self-excited chatter vibrations have been carried out for many years all over the world and were initiated by Tlusty [4] and Tobias [5]. Over the years, many theories and approaches have been developed to suppress the negative phenomenon of self-excited vibrations in machining. Some methods were focused on the process modification in such a way that it remained stable. An example of this method was active vibration elimination, as presented by Parus et al. in [6,7], in which vibration dampers or active components of machine tools were used. Another approach is to use neural networks to predict chatter vibrations, as proposed by Cherukuri et al. in [8,9]. A significant group of research has looked at the methods for searching the machining process stability [10]. In this group of methods, the search for stability took place in the plane of technological parameters—the aim of these procedures was to find spindle speeds and cutting depths where self-excited vibrations did not develop [11]. The basic tool enabling the selection of these parameters were the so-called stability lobes [12,13], showing the cutting depth at which the chatter vibrations would occur as a function of the spindle rotational speed. In order to determine the stability lobes, first, it is necessary to know the dynamic properties of the machine–tool–workpiece, expressed as the frequency response function (FRF). It is possible to determine the FRFs in different ways. In experimental methods, a series of impulse tests are carried out, in which the structure is excited by the modal hammer, whereas the system response is measured by the accelerometers. This is a widely used method [14,15], characterized by high reliability, however, it is necessary to have highly qualified staff and specialized measurement equipment, and the method is time-consuming. Another approach is the modeling of dynamic systems of machine tools using the finite element methods, as presented by Dunaj et al. in [16,17]. However, although these methods achieve good results, they remain computationally complex and require a highly qualified team with extensive experience. An alternative approach is the use of modal synthesis methods such as receptance coupling, which combines the advantages of the experimental and analytical methods [18]. It allows us to determine the dynamic properties of the machine tool system, with an experimental model of the machine tool and an analytically evaluated tool model for milling [19] or a workpiece for turning [20,21]. This approach allows a quick determination of the dynamic properties of the machine tool in industrial applications—the system does not need highly qualified staff or specialized equipment.

Qualified CNC operators should have a wide knowledge in the field of machining technology and G-code programming, particularly in light of the increasing demands on machining time, new methods of surface quality and topographic inspection [22], and continuous miniaturization that require an accuracy range in micrometers [23]. Machining tools are also constantly being developed and extended with modern, complex systems such as, compensation of thermal errors of a ball-screw-driven [24] or a vision-based 3D (three-dimensional) scanning system, for the positioning of the workpiece [25]. Both of these systems complicate the programming process, which requires specific knowledge from the operator. The simplest CNC systems do not have embedded tools to assist the operator in preparing even a machining program. Additional CNC system options provide only graphical interfaces to facilitate G-code generation and analysis (Fanuc–Manual Guide, Siemens–Shopturn/ShopMill). Machine tool manufacturers also offer their own HMI (Human Machine Interface) solutions. Their activities focus on the development of a unified operator interface, independent of the used control system (e.g., DMG Mori–Celos, Mazak–Mazatrol). New graphics solutions with 3D elements, touch screens, remote controls, and even gesture control are being introduced to enhance the operator comfort. However, none of these solutions support the operator in the selection of technological parameters. Currently, in connection with attempts to adapt machines to the idea of industry 4.0, research is being conducted on systems that also support the operator in the selection of technological parameters [26,27,28,29]. The popularity of these systems will keep increasing. This will be facilitated by the increasing computational power of the CNC systems and the growing number of sensors integrated in the machines.

This study presents the implementation process of an algorithm to prevent chatter vibration, using the receptance coupling method in the Fanuc control system. Unlike most common solutions to eliminate vibration, the proposed method is a purely software-based solution, so it does not require costly hardware modification of the machine. In Section 2, the issue of determining the stability of machining is discussed and the process of determining the dynamic properties of the machine tool system using the receptance coupling method is described. The third section presents the idea of the developed assistant for the selection of technological parameters. The assistant implementation process in the lathe with the Fanuc control system is explained in the fourth section. The experimental validation of the proposed solution is described in Section 5 and the conclusions are described in Section 6.

## 2. Turning Stability

### 2.1. Stablility Lobes

**Figure 1:**
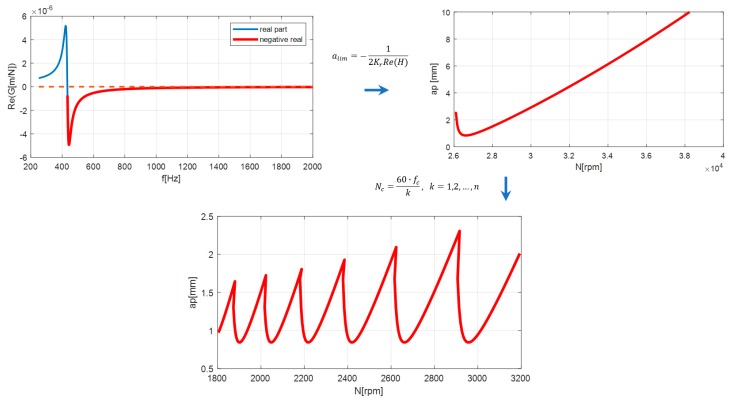
Stability lobes calculation procedure.

Chatter vibration during machining occurs due to loss of stability of the cutting process. It is caused by the relative motion between the machine tool and the workpiece when the tool is cutting previously machined, wavy surface. As a result, a local change of the depth of the cutting layer occurs, which leads to a periodical increase in the cutting force and an increase in vibration amplitudes is observed. The phenomenon is commonly known as regenerative chatter. The parameter that has a significant influence in determining the machining stability is the depth of cut. The limiting depth of the cut $a_{lim}$ for orthogonal turning can be given as [12]:

(1) $$a_{lim}=-\frac{1}{2K_{r}Re\left(G\left(j\omega \right)\right)}$$

where, $K_{r}$ is the cutting force coefficient, Re(G(jω)) is the negative minimum of the real part of the transfer function between the tool and the workpiece. Practically, the transfer function G(jω) is determined for the compliant part of the system, which could be a tool for turning with a long overhang (for boring process) or a flexible workpiece, e.g., slender shafts. The limiting depth of cut calculated using Equation (1) was positive only for negative real part of the transfer function. When the depth of cut was selected under the stability lobe, the process would be stable, providing a smooth machined surface. When the depth of the cut is above the lobe, the process loses stability, with growing vibrations, increasing dynamic forces, and chatter marks on the machined surface. The lobe is replicated for spindle rotational speeds, as follows:

(2) $$N_{c}=\frac{60\cdot f_{c}}{k},\;\;\;\;\;\;\mathrm{for}\;k=1,2,\cdots ,n$$

where $f_{c}$ is the chatter frequency and *k* is positive integer denoting the lobe number, as presented in Figure 1.

The procedure presented in Figure 1 involves the frequency response function of the machine–tool–workpiece system and the cutting force coefficients determine the interaction between the tool and the workpiece material. In order to evaluate the precise limit of the cutting depth, the coefficients should be experimentally determined for each specified tool–workpiece material configuration. In the proposed procedure, the value of the coefficient $K_{r}$ is defined arbitrarily (assuming roughly the conditions of steel cutting), which does not allow the determination of the depth of the cut-limit, however, is still sufficient for optimization of the rotational speed selection.

### 2.2. Receptance Coupling

**Figure 2:**
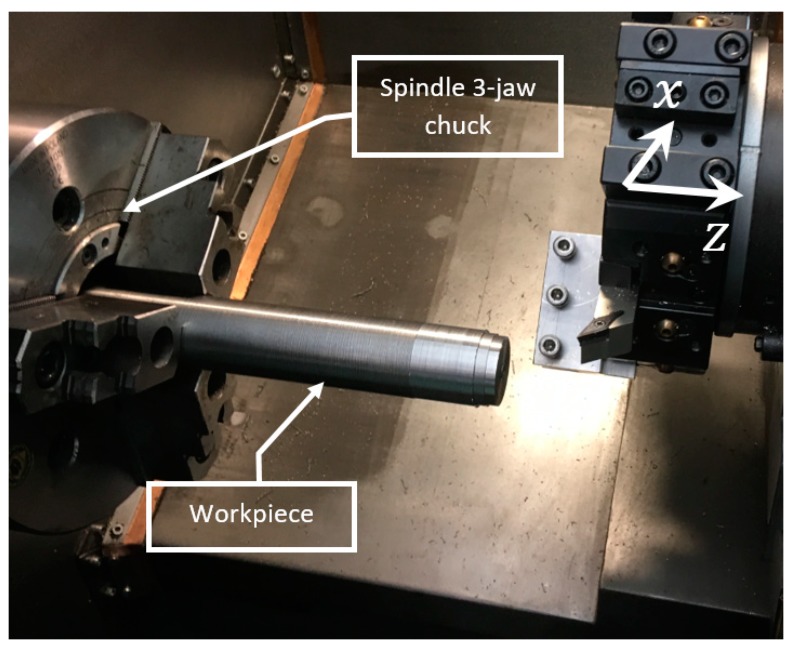
The machine–tool–workpiece system.

Determining the dynamic properties of the machine–tool–workpiece system is carried out using the receptance coupling method. In order to synthesize the dynamic properties of the system, it is necessary to have frequency response functions of its subassemblies. In the presented case, where the workpiece is the compliant part of the system, the components are the rod and the lathe spindle with a three–jaw chuck, as presented in Figure 2.

**Figure 3:**
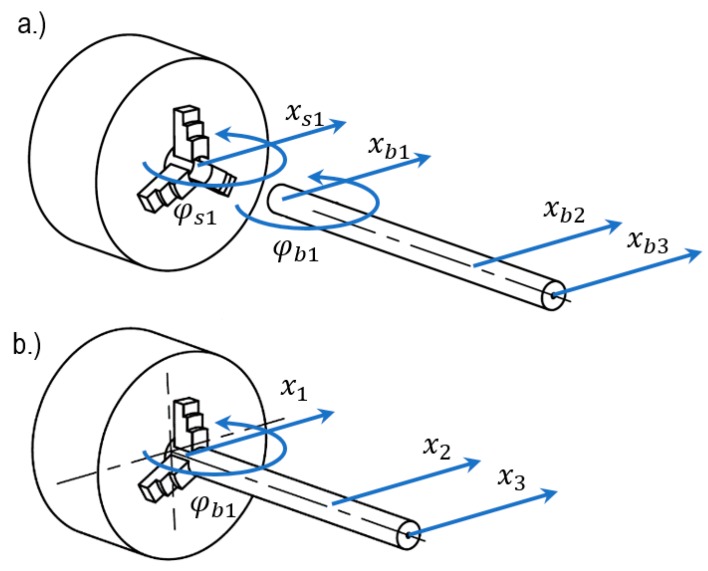
Receptance coupling–system coordinates (**a**) system components; (**b**) coupled system.

The first step in the receptance coupling procedure is to define the dynamic properties of the system components in local coordinates, as presented in Figure 3a.

In the proposed coupling procedure, only the x–direction is considered, as in turning operations, chatter vibrations in this direction has a major impact on the quality of the machined surface. However, the three-jaw chuck fixture results in a bending angle of the spindle in the cutting plane, which causes the same deflection of the workpiece in the coupling point. Therefore, it is necessary to consider the rotational degrees of freedom (RDOFs) of both the spindle and the workpiece at the fixture point. In the workpiece, apart from the coupling point “1”, the additional cutting tool location could be considered in order to evaluate the frequency response function for the stability lobes calculation.

The dynamic properties of the separated system components in local coordinates are expressed in matrix terms, as follows:

(3) $$\left[\begin{array}{c} x_{s1}\\ \varphi_{s1} \end{array}\right]=\left[\begin{array}{cc} H_{s11} & H_{s12}\\ H_{s21} & H_{s22} \end{array}\right]\cdot \left[\begin{array}{c} F_{s1}\\ M_{s1} \end{array}\right]$$

(4) $$\left[\begin{array}{c} x_{b1}\\ \varphi_{b1}\\ \begin{array}{c} x_{b2}\\ x_{b3} \end{array} \end{array}\right]=\left[\begin{array}{cc} \begin{array}{ccc} H_{b11} & H_{b12} & H_{b13}\\ H_{b21} & H_{b22} & H_{b23}\\ H_{b31} & H_{b32} & H_{b33} \end{array} & \begin{array}{c} H_{b14}\\ H_{b24}\\ H_{b34} \end{array}\\ \begin{array}{ccc} H_{b41} & H_{b42} & H_{b43} \end{array} & H_{b44} \end{array}\right]\cdot \left[\begin{array}{c} F_{b1}\\ M_{b1}\\ \begin{array}{c} F_{b2}\\ F_{b3} \end{array} \end{array}\right]$$

where, *x* is the translational displacement, φ is the rotation angle, *F* is the force, *M* is the torque, and *H* is the transfer functions.

For Spindle, described by Equation (3), the determination of the translational transfer function $H_{s11}$ is not problematic and can be easily carried out by performing the impulse tests. However, the experimental determination of the rotational transfer functions turns out to be troublesome. Most of the methods proposed for this type of application turns out to be highly sensitive to measurement inaccuracies or are laborious to achieve good results [30,31]. Therefore, the extended inverse receptance coupling (EIRC) method was used, developed by the author of this paper, specifically for this type of application [32]. The method assumes a determination of the spindle dynamic properties based on experimentally determined frequency response functions of coupled spindle–beam systems. The dynamic properties of the beam are known (can be determined analytically), therefore, as a result of the decomposition of the system using the inverse receptance coupling procedure, the dynamic properties of the spindle itself are obtained. The proposed extension of the method assumes increasing the number of measurement system configurations, which allowed to improve the accuracy of determining the spindle dynamics properties and additionally averaged the joint properties of the workpiece with the spindle chuck jaws.

Workpiece, described by Equation (4), can be modeled analytically as a free–free circular cross-section Timoshenko beam of known length, diameter, and material properties. The proposed procedure requires workpiece translational FRFs in three points, and additionally the rotational FRFs for the point of the fixture in the spindle chuck. The procedure for determining these functions is described in detail by authors in [33].

During the FRFs of the system components, the coupling procedure could be performed. The boundary conditions and equilibrium of forces between the components are imposed and the local coordinates (Figure 3a) are replaced by the global, coupled system coordinates (Figure 3b), as given below:

(5) $$\{\begin{array}{c} x_{S1}=x_{b1}=x_{1}\\ \varphi_{s1}=\varphi_{b1}=\varphi_{1} \end{array},\;\;\;\;\;\;\{\begin{array}{c} F_{S1}+F_{b1}=F_{1}\\ M_{s1}+M_{b1}=M_{1} \end{array}$$

Transforming Equations (4) and (5), the coupled system FRFs matrix is obtained, which could be expressed as a product of the inverse transformation matrix T and the beam matrix:

(6) $$\left[\begin{array}{c} x_{1}\\ \varphi_{1}\\ \begin{array}{c} x_{2}\\ x_{3} \end{array} \end{array}\right]=T\cdot \left[\begin{array}{cc} \begin{array}{ccc} H_{b11} & H_{b12} & H_{b13}\\ H_{b21} & H_{b22} & H_{b23}\\ H_{b31} & H_{b32} & H_{b33} \end{array} & \begin{array}{c} H_{b14}\\ H_{b24}\\ H_{b34} \end{array}\\ \begin{array}{ccc} H_{b41} & H_{b42} & H_{b43} \end{array} & H_{b44} \end{array}\right]\cdot \left[\begin{array}{c} F_{1}\\ M_{1}\\ \begin{array}{c} F_{2}\\ F_{3} \end{array} \end{array}\right]$$

The transformation matrix T containing the spindle, the workpiece, and the tailstocks transfer functions, is given as:

(7) $$T^{-1}=\left[\begin{array}{c} \begin{array}{cccc} 1+\frac{H_{b11}H_{S22}-H_{b12}H_{S12}}{H_{S11}H_{S22}-H_{S12}H_{S21}} & \frac{H_{b12}H_{S11}-H_{b11}H_{S12}}{H_{S11}H_{S22}-H_{S12}H_{S21}} & 0 & 0\\ \frac{H_{b21}H_{S22}-H_{b22}H_{S12}}{H_{S11}H_{S22}-H_{S12}H_{S21}} & 1+\frac{H_{b22}H_{S11}-H_{b21}H_{S12}}{H_{S11}H_{S22}-H_{S12}H_{S21}} & 0 & 0\\ \frac{H_{b31}H_{S22}-H_{b32}H_{S12}}{H_{S11}H_{S22}-H_{S12}H_{S21}} & \frac{H_{b32}H_{S11}-H_{b31}H_{S12}}{H_{S11}H_{S22}-H_{S12}H_{S21}} & 1 & 0\\ \frac{H_{b41}H_{S22}-H_{b42}H_{S12}}{H_{S11}H_{S22}-H_{S12}H_{S21}} & \frac{H_{b42}H_{S11}-H_{b41}H_{S12}}{H_{S11}H_{S22}-H_{S12}H_{S21}} & 0 & 1 \end{array} \end{array}\right]$$

Finally, having the dynamic properties of the machine–tool–workpiece system, a stability analysis could be performed.

## 3. The Assistance of Machining Parameters Selection

The standard manufacturing process involving the CNC machine tool, at first, requires technical drawing. Then, the machining program is implemented on a specific machine by the programmer. The program includes instructions for a toolpath to cut away the material of the workpiece to obtain the geometry of the machined part and technological parameters (feedrate, depth of cut, cutting speed, or the resultant spindle speed). The selection of these parameters is based on the knowledge of the machine technology, the experience of the programmer, and the ranges of the parameters recommended by the tool insert manufacturer. Then, the program through the CNC control system activates the spindle drive, feed drives, etc., in order to carry out the cutting process. However, in the presented standard approach, the dynamic properties of the machine–tool–workpiece system are not considered, therefore, there is a risk of self-excited chatter vibrations.

**Figure 4:**
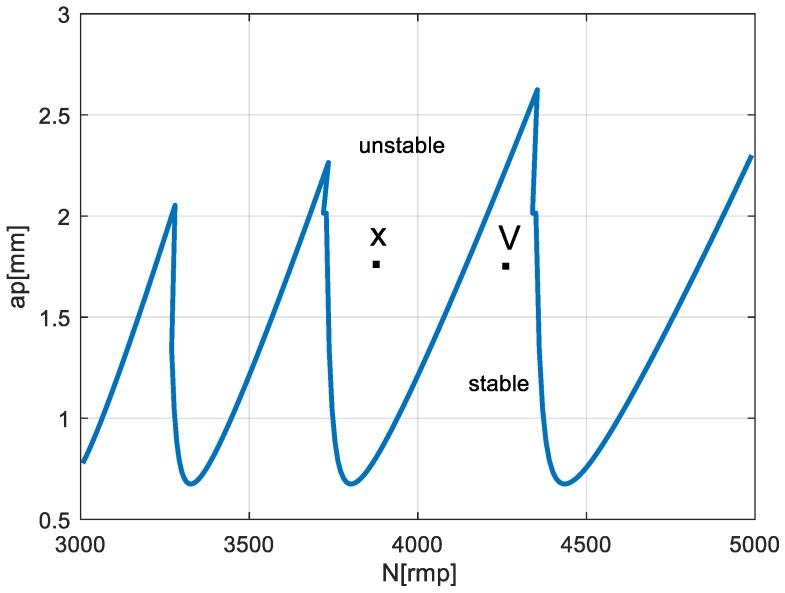
Cutting-speed selection problem in a stability lobes diagram.

The problem of selecting the appropriate rotational speed can be presented on the example of the turning the shaft with a 30 mm diameter. For a cutting tool with an insert VBMT 16 04 08–PR 4325 (Sandvik Coromant, Sandviken, Sweden), the cutting speed range supplied by the manufacturer (335–450 m/min), for the defined cutting diameter gives a wide range of recommended rotational spindle speed (approximately 3550–4770 rpm). In a standard approach, the speed for machining program is usually selected arbitrarily. However, when considering the dynamic properties of the system, setting some speeds for a specific cutting depth would provide a stable cutting, while for others, chatter vibration would occur. This problem is presented in an example stability lobes diagram in Figure 4.

However, the use of stability lobes in industrial practice, in its current form, is troublesome, due to the need to carry out experimental research for each machine–tool–workpiece configuration. Apart from the fact that this is time-consuming to perform, it also requires a specialized equipment and software.

**Figure 5:**
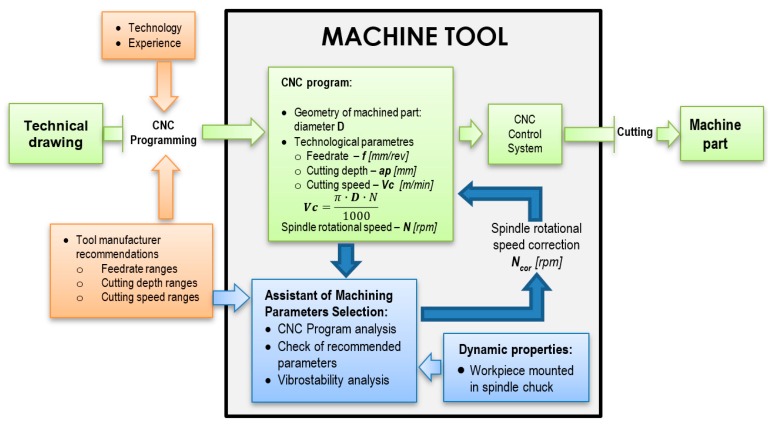
Algorithm of the Assistant of Machining Parameters Selection.

The proposed Assistant of Machining Parameters Selection is an internal application installed in the CNC system. First, it requires entering the ranges of the machining parameters recommended by the tool manufacturer or it is based on the user experience. Then, the dynamic properties of the machine–tool–workpiece system using the receptance coupling procedure are calculated. Ultimately, the procedure is implemented as part of the assistant, however, in the development version it is performed externally. Based on the dynamic properties, the stable cutting speeds are calculated. The analysis of the CNC program consists of checking the applied spindle speeds and comparing them with the set of “stable” speeds available. Then, an obligatory speed correction is proposed. In those cases where it was chosen arbitrarily and could be changed, this correction is highly recommended. The algorithm of the presented Assistant of Machining Parameters Selection is shown in Figure 5.

In the presented diagram, the standard machine programming path without the use of the assistant is highlighted in green. It should be noted, that the implementation of the additional procedures (highlighted in blue) does not change this approach but is only an additional feature. As a result, the operator receives additional functionality of the machine, without having to change existing habits.

## 4. Implementation of the Algorithm in the CNC System

### 4.1. Operator Assistance Systems

Nowadays, manufacturers of CNC control systems and machine tool manufacturers offer their own graphical interfaces to facilitate the generation of G-code: Fanuc–Manual Guide, Siemens–Shopturn/ShopMill, Mazak–Mazatrol, Mitsubishi–Navi Mill, Okuma–OSP, DMG Mori–Celos, etc. These solutions simplify the generation of the G-code, but do not assist the operator in the choice of machining parameters.

Academic centers are also engaged in research on algorithms and solutions automating the selection of the processing parameters. Commonly used methods for chatter suppression is to alter the dynamics of machine tools by using additional passive or active devices to expand the chatter boundary. This approach was presented by Paul and Moralez–Mendelez [34]. Authors use PD/PID regulators (PD—Proportional Derivative, PID—Proportional Integral Derivative) and fuzzy logic algorithms to control active vibration damper installed on the top of the spindle. A similar approach based on the same method was developed by Wan [35] and Fallah [36]. Researches integrated a non-contact electromagnetic actuator with two degrees of freedom into a specially designed compact spindle system. Piezoelectric sensors such as active suppression actuators was used by Tang et al. [37] and Liu [38]. In both studies, the researchers integrated the piezoelectric patches for controlling the vibration and chatter phenomenon in the boring bar. A passive suppression device mounted on the boring bar was proposed by Miguélez [39] and a device mounted in the boring bar was developed by Yang [40]. Most of these systems, despite their effectiveness, could not be used for serially produced machines. Active solutions based on piezoelectric or electromagnetic actuators require significant and costly reworking of the spindle construction, the bearing system, or the mechanical construction. Moreover, after actuators are embedded in the machine–tool construction, they require additional power supplies, data acquisition cards, and control system (especially piezoelectric actuators). The cost of reconstructing the machine structure and purchasing additional electronic systems is economically unjustified for machine–tool manufacturers. In addition, implementing a complex control system in a tool is difficult due to cable connections. The tools also wear out during machining and could be damaged by collision or during exchange by the operator.

The system described in this study is free from the drawbacks of the systems described above. The proposed solution could be implemented in any control system, does not require any additional hardware to operate, and can be used by the operator after a short instruction.

### 4.2. Implementation in FANUC CNC System

Vibration during assistance of machining parameters selection system has been implemented in the medium size lathe AFM TAE 35 “Hanka” (AFM DEFUM S.A. Andrychów, Poland). The lathe was produced by the Andrychowska Fabryka Maszyn DEFUM S.A. FANUC CNC 31i control system; model B was installed in the machine.

Microsoft Visual Studio (2017, Microsoft, Redmond, United States), Fanuc Picture (v3.7.2, Fanuc, Oshino, Japan), Fanuc NCGuide (v12.1, Fanuc, Oshino, Japan) and the Fanuc CNC application development kit (v01.3, Fanuc, Oshino, Japan) with special C++ libraries were used for the implementation. Since the CNC control system controller was based on a PowerPC processor, it was necessary to use the Wind River Diab Compiler for PowerPC.

**Figure 6:**
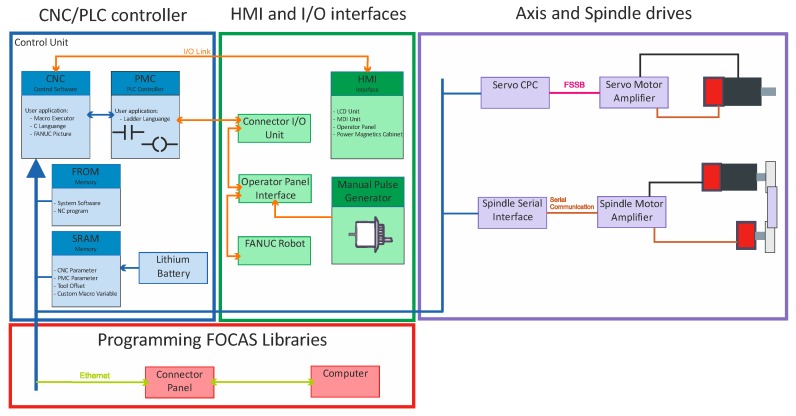
Structure of the Fanuc CNC control system.

The FANUC Picture software is a development environment for creating customized operator screens. It allows to implement complicated machining processes and provides all the functions and features necessary to modify and create modern HMI screens. The developed screens were compiled and stored in the CNC Flash–ROM (FROM) memory and were rendered directly by the CNC processor, without using an additional computer or controller. The use of additional libraries C++ allowed for the direct implementation of the machining parameters selection algorithm. These libraries were required to create custom functions that were not available in the CNC system. C++ functions allows direct read/write access to variables, parameters, and registers in the PMC (Programable Machine Control) or PLC (Programable Logic Controller) memory; control of the machine axis; perform complex calculations; and connect the external devices. In the solution development stage, it was also necessary to use the NCGuide CNC simulator. NCGuide simulator is a software that runs on a PC (Personal Computer), and provides authentic part programming, operation, and a maintenance environment without using a production machine tool; supports both conventional G-code part programming; and conversational part programming. Using this software, users can create and edit part programs; generate cycle time estimates; and create test and debug custom macro subroutines. The complete architecture of the Fanuc CNC control system is shown in Figure 6.

Prior to the implementation of the assistance system, the following assumptions were made:

- the system must be implemented directly in the CNC control system,
- the system cannot change the existing functionality of the control system
- the system should provide intuitiveness by limiting the amount of data entered by the operator

During the development of the HMI system, all assumptions were achieved. Two user interface screens were prepared. The prepared screens showed the startup screen, allowing the user to call up the parameter input screen or to switch to other machine functions. This screen was called automatically after the machine started. In order to use the system, the operator needs to enter all parameters necessary to calculate the recommended machining parameters in the input boxes. At this stage, the operator needs to specify the workpiece dominant mode frequency, diameter of the workpiece, the recommended cutting speed range, and the chosen cutting speed; the proposed cutting speeds were then displayed as output. Then, it is necessary to modify the corresponding values in the G-code. In the first system version, the operator needs to enter all parameters required to calculate the recommended machining parameters, but in the next version of the proposed solution, the number of parameters entered by the operator is reduced. The diameter of the workpiece and the selected cutting speed is extracted from the machining program (G-code). Next, the appropriate algorithm changes the cutting speed value.

## 5. Experimental Validation

**Figure 7:**
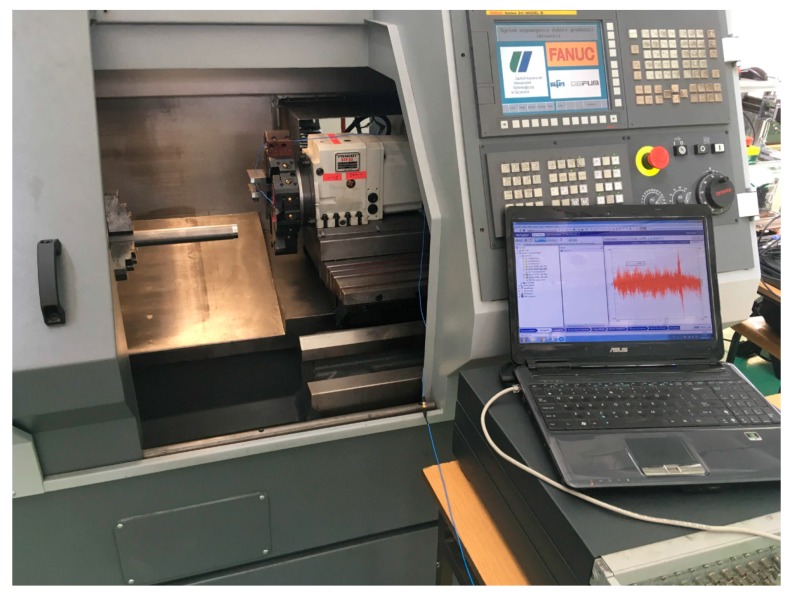
Experimental stand–TAE 35N.

The next part of the presented research is the validation of the proposed solutions. For this purpose, the assistant was implemented on an AFM TAE 35 “Hanka” CNC lathe. The considered workpiece mounted in the three-jaw spindle chuck was a rod of diameter D = 35 mm and length L = 210 mm, made of steel A10x (1.0715). The SVJCL 2020–16 (Pafana S.A., Pabianice, Poland cutting tool was used, equipped with a VCMT 160402–SM (Iscar Ltd., Migdal, Israel) cutting insert (35° rhombic 7° positive flank, nose radius 0.2 mm, with TiAlN+TiN coating layer). In order to detect the occurrence of chatter vibrations, during machining, the vibration was measured using the LMS Scadas data acquisition system with the piezoelectric accelerometer PCB 356A01 (PCB Piezotronics, Depew, NY, USA) with sensitivity 5 mV/g ± 20%. The experimental stand is presented in Figure 7.

**Figure 8:**
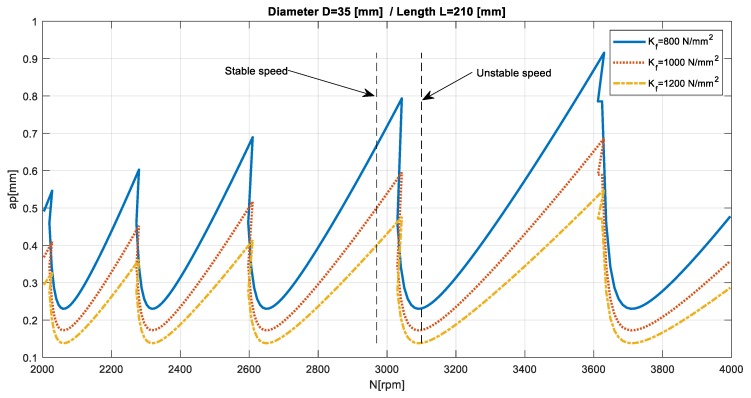
Stability lobes calculated for the machine–tool–workpiece system.

First, the dynamic properties of the machine–tool–workpiece was evaluated using the receptance coupling procedure. The first identified vibration mode was at a frequency of 299 Hz. Then, the cutting stability analysis was performed. The stability lobes for the experimental system are presented in Figure 8.

As previously noted, the proposed procedure did not require defining the cutting force coefficients, because the goal was not to precisely determine the cutting depth limit, but only to indicate a “stable” speed. Figure 8 presents the dependence of the stability lobes on the selected cutting force coefficient. It could be seen that this factor significantly determined the “stable” depth of cut, while it negligibly influenced the choice of a “stable” rotational speed.

**Figure 9:**
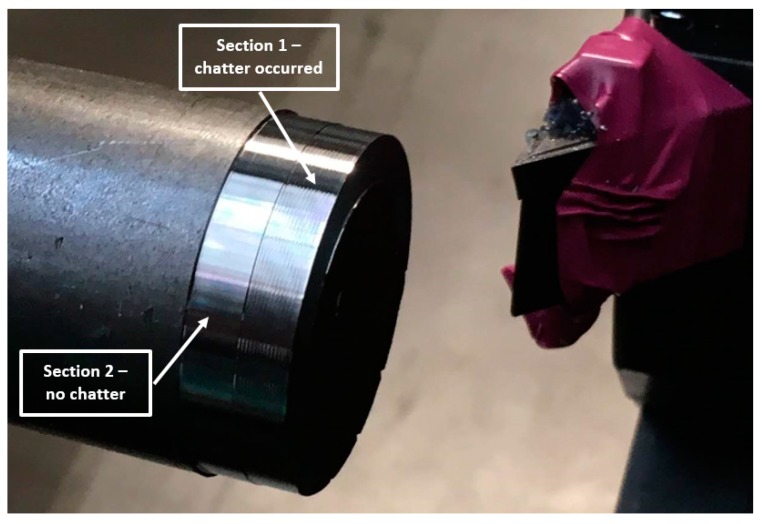
Experimental test “1” results—machined surfaces.

The experimental validation of the effectiveness of the assistant’s involved the machining of two 5-mm sections of the workpiece at its end. The first section, closer to the face, was pre–machined in order to allow further machining on both sections with the same cutting depth, without going through the already machined-surface. The cutting depth for both sections was $a_{p}=0.5\;\mathrm{mm}$ and feed $f_{n}=0.5\;\mathrm{mm}/\mathrm{rev}$. The first section was machined with an arbitrarily set cutting speed of 330 m/min, which for the diameter of 34 mm gave a rotational speed of approximately N = 3100 rpm, while the second section was machined with the spindle speed recommended by the assistant N = 2950 rpm. The machined surfaces are presented in Figure 9.

**Figure 10:**
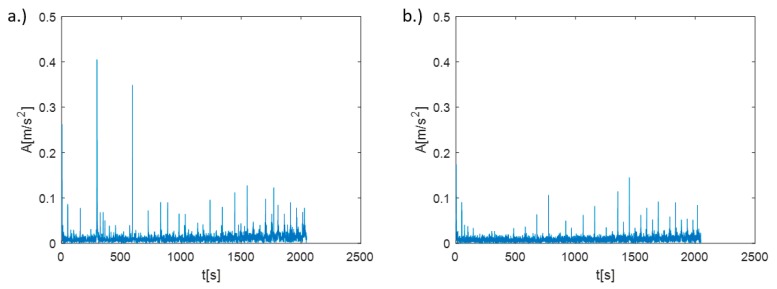
The FFT of vibrations measured during the machining, test “1”: (**a**) Section 1, (**b**) Section 2.

Analyzing the stability lobes presented in Figure 8, it could be stated that for the presented machine–tool–workpiece system, the chosen speed N = 3100 rpm was not suitable. However, in the standard industrial conditions, the stability lobes were not calculated. As a result, despite setting the cutting speed as recommended by the tool manufacturer, chatter marks could be observed on the surface of the first machined section and were registered. The machining of the second section was carried out with the spindle rotational speed determined by the assistant of the machining parameters selection. Machining at N = 2950 rpm, with the same cutting depth and feed, allowed the obtainment of a clean surface without chatter marks. The FFT (Fast Fourier Transform) analysis of vibration measured during the machining also confirmed the occurrence of chatter vibration in Section 1 (Figure 10a) revealing the frequency peak at 295 Hz and its subsequent harmonics (590 Hz, 885 Hz), and their absence in Section 2 (Figure 10b).

**Figure 11:**
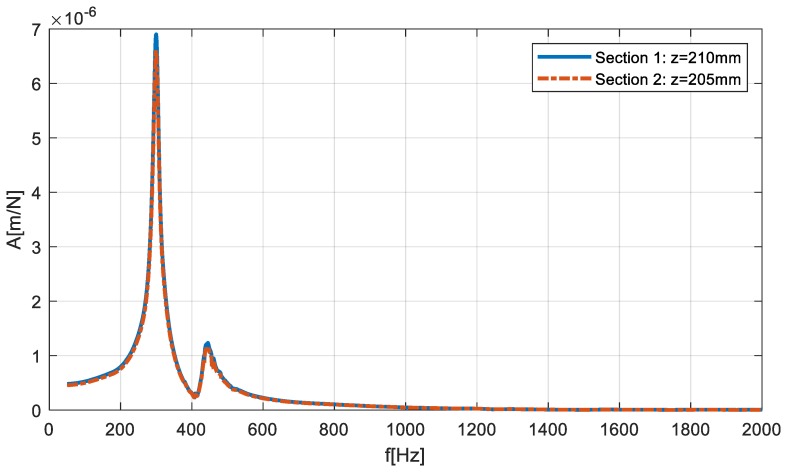
FRFs comparison of the considered sections.

The distance between the two sections was relatively small, however, Section 1 had a higher dynamic compliance, as presented on the FRFs of the machine–tool–workpiece system, determined using the receptance coupling procedure; Figure 11.

**Figure 12:**
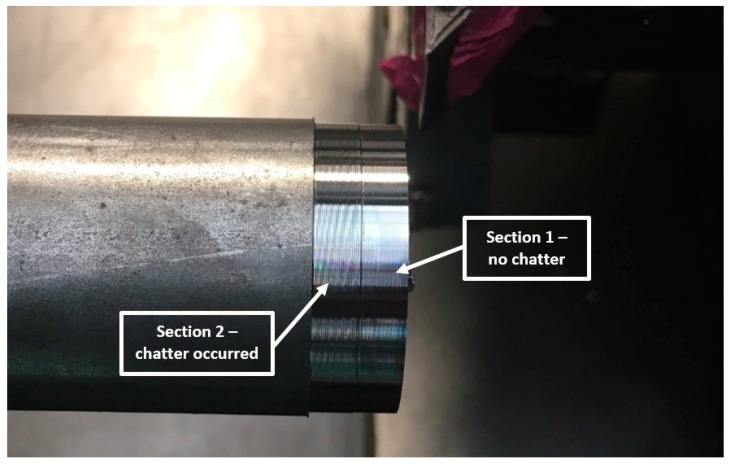
Experimental test “2” results—machined surfaces.

**Figure 13:**
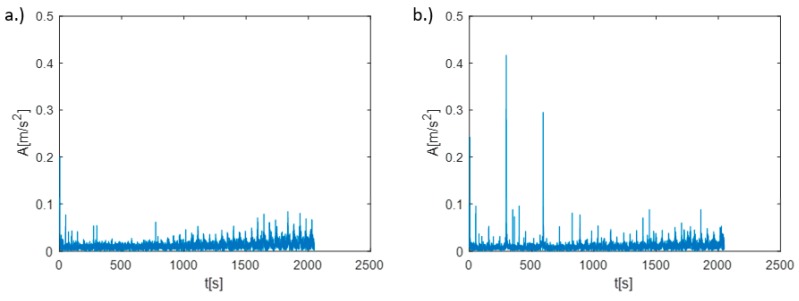
The FFT of vibrations measured during the machining, test “2”: (**a**) Section 1, (**b**) Section 2.

Although (as presented in Figure 11) the differences between FRF of both sections were negligible, it was decided to carry out an experiment in which the first section was machined at a “stable” speed of N = 2950 rpm, while the second section was machined at speed a of N = 3100 rpm where a loss of stability occurred. Again, machining was carried out with the same depth of cut and feed for the same workpiece (mounted in the spindle chuck after changing sides). The obtained machined surfaces are shown in Figure 12, while the FFT of the measured vibration is in Figure 13.

Both, the analysis of the surface quality of the machined surface and the FFT of vibrations measured during machining indicated the occurrence of chatter vibrations in Section 2, where the speed was defined arbitrarily, while in Section 1, where the machining was carried out at the speed proposed by the assistant, no chatter vibrations were observed.

## 6. Conclusions

This study presented the problem of selecting cutting parameters while machining compliant workpieces. In industrial practice, the choice of cutting parameters is based on the operator experience and the ranges specified by the tool manufacturer. For rigid workpieces, the parameter values only determine the relationship between the production efficiency and tool wear rate. However, for the compliant parts, they also determine the occurrence of vibrations during machining. In order to facilitate this task, an approach was proposed in which the selection of technological parameters is supported by an additional program integrated in the CNC control system. The dynamic properties of the machine–tool–workpiece system are evaluated using the receptance coupling method, so that there is no need for expensive, specialized, measuring equipment. As a result, the algorithm proposes parameters correction to ensure stable cutting conditions. The conducted experimental research indicates the beneficial effect of the use of the assistant to support the selection of the spindle rotational speed. However, it should be noted that this is only a software solution that contains some simplifications, therefore, it would not always help avoid chatter vibrations during machining. For workpieces characterized with too high a compliance, the algorithm would not be helpful, however, as the study showed, in some situations it could easily provide stable machining. It should also be emphasized that the proposed solution could be applied not only to new machine tools but also to existing ones, which would increase their capabilities without expensive hardware modifications.

Future research will include a full implementation of the receptance coupling procedure in the CNC control system. In the improved system, parameters describing the geometry of the workpiece will be obtained from the machining program using the G–code.
